# Fall-Related Psychological Concerns and Anxiety among Community-Dwelling Older Adults: Systematic Review and Meta-Analysis

**DOI:** 10.1371/journal.pone.0152848

**Published:** 2016-04-04

**Authors:** Marie-Christine Payette, Claude Bélanger, Vanessa Léveillé, Sébastien Grenier

**Affiliations:** 1 Department of Psychology, Université du Québec à Montréal, Montreal, Quebec, Canada; 2 Centre de recherche de l’Institut Universitaire de Gériatrie de Montréal, Montreal, Quebec, Canada; 3 Laboratoire d'Étude sur l'Anxiété et la Dépression gÉRiatrique (LEADER), Montreal, Quebec, Canada; 4 Department of Psychiatry, McGill University, Montreal, Quebec, Canada; 5 Department of Psychology, University of Montreal, Montreal, Quebec, Canada; University of Geneva, SWITZERLAND

## Abstract

Fear of falling and other fall-related psychological concerns (FRPCs), such as falls-efficacy and balance confidence, are highly prevalent among community-dwelling older adults. Anxiety and FRPCs have frequently, but inconsistently, been found to be associated in the literature. The purpose of this study is to clarify those inconsistencies with a systematic review and meta-analysis and to evaluate if the strength of this relationship varies based on the different FRPC constructs used (e.g., fear of falling, falls-efficacy or balance confidence). A systematic review was conducted through multiple databases (e.g., MEDLINE, PsycINFO) to include all articles published before June 10^th^ 2015 that measured anxiety and FRPCs in community-dwelling older adults. Active researchers in the field were also contacted in an effort to include unpublished studies. The systematic review led to the inclusion of twenty relevant articles (n = 4738). A random-effect meta-analysis revealed that the mean effect size for fear of falling and anxiety is r = 0.32 (95% CI: 0.22–0.40), Z = 6.49, p < 0.001 and the mean effect size for falls-efficacy or balance confidence and anxiety is r = 0.31 (95% CI: 0.23–0.40), Z = 6.72, p < 0.001. A Q-test for heterogeneity revealed that the two effect sizes are not significantly different (Q(19) = 0.13, p = n.s.). This study is the first meta-analysis on the relationship between anxiety and FRPCs among community-dwelling older adults. It demonstrates the importance of considering anxiety when treating older adults with FRPCs.

## Introduction

Fear of falling and other fall-related psychological concerns (FRPCs) (e.g., falls-efficacy or balance confidence) are highly prevalent among community-dwelling older adults, with prevalence generally ranging from 21% to 85% across studies [1]. Fear of falling and falls-efficacy or balance confidence have different theoretical origins. Fear refers to a temporary state of apprehension towards an explicit threat (in this case, the threat is a fall), while self-efficacy (or falls-efficacy) refers to one’s confidence in its ability to manage a threat, such as falls [2,3]. FRPCs therefore refer to a group of distinct but related constructs: self-efficacy is a resilience factor that may influence the level of fear experienced in the face of a threat (e.g., “I am very confident that I will not fall, therefore I am not afraid of falling”) [3,4]. While some community-dwelling older adults with FRPCs have many risk factors for falls, some others do not, and their FRPCs seem excessive, considering that their actual fall risk is low [5,6]. Excessive concerns may take the form of an anxiety disorder, and since 2013, the *specific phobia* section of the Diagnosis and Statistical Manual of Mental Disorders (DSM-5) [7] addresses fear of falling among elderly. In addition, some authors report that individuals with FRPCs tend to have more general worries, such as forgetting an appointment or getting robbed [8]. These symptoms may reveal excessive anxiety or worry about a number of themes, which may suggest the presence of a Generalized Anxiety Disorder (GAD) [7,9]. An excessive reduction of activities caused by FRPCs is likely to have deleterious consequences for the health and the well-being of an older adult, such as loss of autonomy, isolation, and a decrease in physical condition, which can than lead to an increased risk of falls [10]. A recent meta-analysis [11] qualified current evidence as “low” for the reduction of FRPCs through exercise programs only. This underlines the need for a better understanding of the role of psychological factors, such as anxiety, to help adapt interventions to the psychological profiles of community-dwelling elderly with FRPCs.

It would appear intuitive that FRPCs would be associated with anxiety. However, when examining the literature on FRPCs and anxiety, a recent systematic review conducted on studies published between 2006 and 2013 concluded that results were mixed and evidence was insufficient to render conclusions on this relation [12]. In addition, fear of falling is often used as an umbrella term [13] and many authors use the terms “falls-efficacy” and “fear of falling” interchangeably [4]. In their systematic review, Denkinger, Lukas, Nikolaus and Hauer [12] found that the two constructs do not necessarily appear to relate similarly to anxiety; while anxiety was generally related with falls efficacy in the literature, it was not the case with fear of falling. Another review also concluded that evidence of a significant relationship between anxiety and FRPCs is insufficient [14], but the inclusion of studies on populations with special medical conditions (e.g., chronic obstructive pulmonary disease) and the comparison of results from bivariate associations with results from multivariate associations rendered the review’s conclusions difficult to interpret. None of these two reviews formally assessed the quality of the included studies, which is an important step for interpreting the results in light of the various limitations of each study. Further, both reviews based their conclusions on comparing the number of studies presenting significant and non-significant results. Borenstein, Hedges, Higgins and Rothstein [15] warn against the interpretation of results obtained through this method, which they refer to as “vote counting”, since it does not account for the power of the studies (i.e., their ability to detect significant results). Therefore, Borenstein et al. [15] strongly recommend the use of a meta-analysis to allow a rigorous interpretation of the quantitative results issued from a systematic review.

The purpose of the present study is to provide a quantitative summary of the relationship between anxiety and FRCPs among the community-dwelling older adults. Moreover, this study aims at evaluating if the strength of this relationship varies depending on the construct used. It will answer the following question: Are anxiety symptoms differently related to 1) fear of falling and 2) falls-efficacy or balance confidence?

## Methods

### Search strategy and selection criteria

This review was conducted following the PRISMA guidelines for systematic reviews and meta-analysis [16]. An electronic search identified published articles measuring fear of falling (or falls-efficacy or balance confidence) and anxiety among community-dwelling elders. The search included all articles published in English or in French before June 9^th^, 2015 and was conducted in the following databases: PsycINFO, Web of Science, MEDLINE (with FullText (EBSCO) (XML)), Scopus, PubMed, and CINAHL. For PsycINFO, the full search strategy was the following: Anxiety (abstract) AND (“Fear of falling” or “Falls efficacy” or “Balance confidence” or “Ptophobia” or “Fall-related concern”) (abstract). The full search criteria for all databases are presented in S1 File. The search and selection of articles, in which titles, abstracts, and full-text articles were examined for inclusion, were conducted independently by two reviewers. Disagreements were resolved through discussion and obtainment of a consensus, or by consultation of a third person (SG). Articles were included if they met the following criteria: Mean age of participants above or equal to 65 years old, participants living in the community, and if they included measures of FRPCs and anxiety. Measures of anxiety had to be general, meaning that no article reporting measures of a specific anxiety disorder, such as agoraphobia, panic disorder, post-traumatic disorder, and specific phobia was retained. This was done in order to avoid grouping different studies, which could threaten the validity of the meta-analysis [17]. However, since many non-specific anxiety symptoms questionnaires can also be used to screen for GAD (e.g., Geriatric Anxiety Inventory, Hamilton Anxiety Rating Scale, Hospital Anxiety and Depression scale, etc.), we considered these questionnaires to be suitable for inclusion. This was also done by two recent systematic reviews [12,14]. Articles were excluded if the study was solely conducted on a population affected by specific medical characteristics (e.g., Parkinson’s disease, stroke, chronic obstructive pulmonary disease, arthritis or dizziness) or if the design was a case study. Following the identification of potentially relevant titles, abstracts and full-text articles were screened. If two articles or more reported data from the same study, the article presenting more details pertaining to sample characteristics and measures of FRPCs or anxiety was selected. After having selected all relevant articles, a manual search was conducted through their reference list: The same selection criteria and procedure, including the manual reference search, were applied, until no more new articles were found. This search strategy implied that articles measuring anxiety and FRPCs were first identified even if they did not report the association between those two variables. If the article reported measures of anxiety and of FRCPs, the authors were contacted to see if they were able to provide data regarding the bivariate association (e.g., correlations, differences between means) or supplementary details required for assessing inclusion or exclusion criteria (e.g., if participants were community-dwelling). This procedure was followed in an effort to include unpublished data. Twelve authors were contacted for additional information; one more author was not contacted because the email address was not valid. Six authors were able to provide the information; the six others did not have the requested information or did not reply. In addition, six active researchers in the field of FRCPs were contacted to inquire if they had conducted any unpublished studies in which the association between FRCPs and anxiety was measured: This inquiry led to the inclusion of one new study. This last study had been published, but analyses concerning FRPCs and anxiety were not included in the article and were provided separately (by Kim Delbaere, Ph.D. and Stephen Lord, Ph.D.) for the purpose of this meta-analysis.

**Fig 1:**
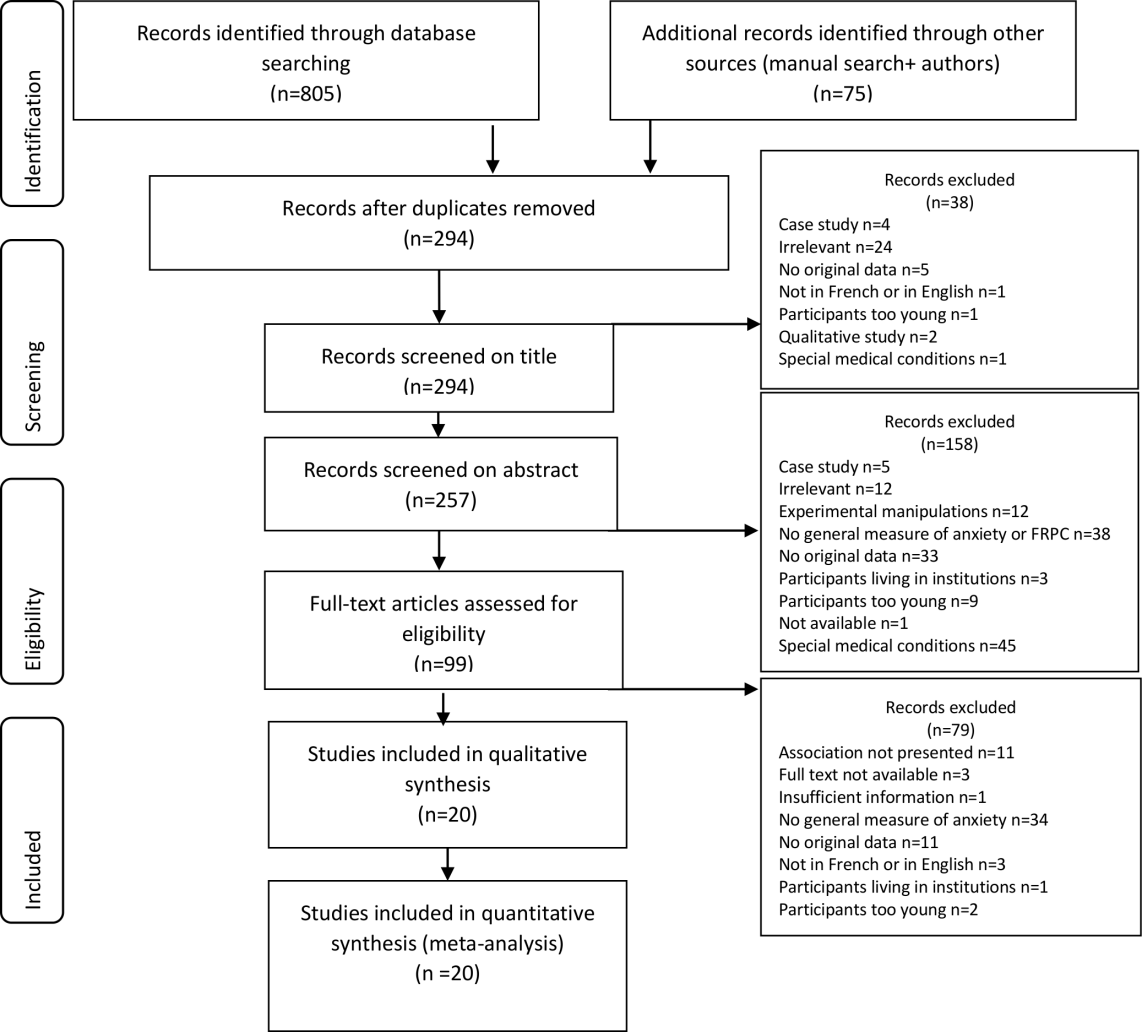
PRISMA flow diagram of the inclusion of studies.

The database searches identified 820 articles. Seventy-four other articles were identified through manual searches and one article came from contacting active researchers of the field (as mentioned above). A total of 294 articles remained after duplicates were removed (see Fig 1). After screening through titles (n = 294), abstracts (n = 257), full-text articles (n = 99), and contacting authors for supplementary information, 20 different studies were selected (see Fig 1).

### Data extraction

The following information was extracted from the selected studies: country where the study was conducted, number of participants, age of participants (mean, standard deviation or range), rating scale for FRPCs, rating scale for anxiety, bivariate associations between FRPCs and anxiety (e.g., correlations, difference between means). If measures were taken repeatedly (e.g., pre-test/post-test measures in the case of intervention studies), only the pre-test measures were extracted. Data was extracted independently by two authors in a pre-designed form and results were compared and verified by both authors.

### Classification of FRPCs

Classification of scales measuring FRPCs in either one the following categories: 1) fear of falling and 2) falls-efficacy or balance confidence, was based on a review by Jorstad, Hauer, Becker and Lamb [18]. Effects sizes were later calculated for each of the two categories.

The first category encompasses scales measuring fear of falling and comprises the following: Mobility Efficacy Scale (MES) [19], adapted Falls Efficacy Scale (aFES) [19], Survey of Activities and Fear of Falling in the Elderly [SAFFE (or SAFE)] [20], and University of Illinois at Chicago Fear of Falling Measure (UIC FFM) [21]. The Falls Efficacy Scale–International (FES-I) [22], was also added to this category, since it was published after Jorstad et al.’s review [18]; this scale measures concerns about falls (although its name suggests that it measures falls-efficacy). In addition to these scales, this category also includes single-item measures, such as “Are you afraid of falling?”, “Are you concerned about falls?”, as proposed by Jorstad et al. [18].

The second category encompasses scales measuring self-efficacy or balance confidence or confidence at avoiding falls. Those scales were grouped based on Jorstad et al.’s findings [18] and a psychometric study by Hotchkiss et al. [23]. This category includes the Falls Efficacy Scale (FES) [24], the Falls Efficacy Scale revised (rFES) [25], the modified FES (mFES) [26], the FES United Kingdom (FES-UK) [27], the Activities-specific Balance Confidence (ABC) [28], the ABC United Kingdom (ABC-UK) [27] and the Confidence in maintaining balance scale (ConFBal) [29].

### Quality assessment

Quality of the selected studies was independently assessed by two reviewers. The criteria used were inspired and adapted from Loney, Chambers, Benett, Roberts and Stratford and Sanderson, Tatt and Higgins’s criteria for evaluation of observational studies [30,31]. The following 10 criteria were selected by the two reviewers: (1) Are the recruitment sources described? (2) Are the criteria for exclusion or inclusion well defined? (3) Are required sample size calculations presented? (4) Is the evaluation procedure clearly described? (5) Has the scale used to measure FRPCs been validated for the population under study? (6) Has the scale used to measure anxiety been validated for the population under study? (7) Are the FRPCs classified appropriately (i.e., Are fear of falling/falls-efficacy or balance confidence measurement scales identified as such?) (8) Are the statistical analysis for evaluation of the association between FRPCs and anxiety described? (9) Does the results section present a minimum of descriptive information about the participants, such as age (mean, range or standard deviation) and gender?, and (10) Are the study's limitations presented? One point was awarded for each criteria. Studies meeting at least 5 of the 10 criteria were judged as being of sufficient methodological quality to be included in the meta-analysis.

### Statistical analysis

For each selected study, correlation coefficients between FRPCs and anxiety were extracted. If the data was presented in the form of mean differences, Cohen’s d was calculated and converted into Pearson correlation, following Borenstein et al. ‘s method [15].

A random-effect model was used to compute effect sizes for 1) the association between fear of falling and anxiety and 2) the association between falls-efficacy (and balance confidence) and anxiety. Effect sizes were calculated with Fisher’s Z transformation and its variance, following Borenstein et al.’s recommendations [15]. Fisher’s Z transformation applies no corrections to effect sizes, but its variance calculations provide an adjustment for sampling error [15]. Borenstein et al. [15] favor this method over Schmidt’s, which applies corrections at the study-level for potential methodological errors [32]. These corrections were not necessary for our objective, which is to report the data as it can be found in the literature [33]. In-between study variance was calculated with the method of moments, as proposed by Borenstein et al. [15]. Results were transformed back into correlation coefficients for interpretation.

For each of the two effect sizes, heterogeneity was assessed with the Q statistic and the I^2^ statistic, which assesses heterogeneity without accounting for the number of studies. The possibility of a publication bias was assessed via a funnel plot of each study’s standard error and correlation for each of the two effect sizes and Rosenthal’s fail-safe n [34]. This last method is controversial because its calculations involve estimating the effect sizes of unpublished studies [15,35]. In other words, the Rosenthal’s fail-safe n measures how many studies with an effect size of “X” would be required for the p-value to be non-significant. Although the choice of this estimated effect size is arbitrary, it provides a way of quantifying the likelihood of a potential publication bias. In order to be conservative, we chose an effect size of zero for our calculations. A Q-test for heterogeneity was conducted to verify if the two summary effects [1] Mean effect size of relationship between anxiety and fear of falling; 2) Mean effect size of relationship between anxiety and falls-efficacy or balance confidence] were significantly heterogeneous [15]. Statistical analyses were performed with Statistical Package for Social Sciences (SPSS) and Lipsey and Wilson’s macro [36].

## Results

### Characteristics of selected studies

The 20 selected studies include 4738 community-dwelling participants and were published between 1990 and 2015. The average age of participants is 77.12 years. Five studies were conducted in Europe [37–41], seven in the USA [24,42–47], three in Canada [48–50], two in Australia [5,51], one in Hong Kong [52] and two in Israel [53,54]. Ten studies measure fear of falling and ten studies measure falls-efficacy or balance confidence (see Table 1).

**Table 1:** Summary of selected studies.

*Study*	*Sample size*	*Age (Mean)*	*FRPC scale*	*FRPC mean or prevalence*	*Anxiety scale*	*Anxiety mean or prevalence*	*Correlation between anxiety and FRPC*	*Quality (/10)*
***Fear of falling***								
Liu 2015 [52]	445	NA	Chinese FES-I	M = 30.44/64	Chinese GAD-7	M = 4.43/21	0.24	6
Greenberg 2015 [46]	107	77	FES-I	M = 38.02/64	Diagnosis with ICD, Y/N	Y = 18	0.68	9
Zijlstra et al. 2013 [41]	120	77.7	Short FES-I	M = 13.7/28	HADS-A	M = 6.5/28	0.47	7
Painter et al. 2012 [42]	99	73.71	SAFE-B	n = 38	HARS	M = 10.54/30	0.40	9
Delbaere et al. 2010 [5]	500	77.9	FES-I	M = 22,51/64	GAS	M = 0.92/9	0.15	9
Smith et al. 2010 [43]	46	83.5	SAFE	M = 0.52/3	STAI—trait	M = 32.3/80	0.00	9
Kempen et al. 2009 [39]	540	77.6	Are you afraid of falling? 5-point Likert scale	Severe fear = 242/540	HADS-A	M = 7.4/28	0.21	7
Murphy et al. 2002 [47]	1064	79.6	Are you afraid of falling? Y/N	Y = 506	STAI ≥32 = Y	Y = 526	0.26*	8
Drozdick et al. 2001 [44]	30	74.35	Single question on severity of FOF (5-point Likert scale)	High fear, n = 18	STAI–trait, cut-off of ≥40	High level = 10	0.41	8
Downton et al. 1990 [40]	203	Median = 83	Do you limit your activities because of FOF? Y/N	Y = 86	Anxiety subscale of GHQ-28	M = 3.39/7	0.26	6
***Falls-efficacy or balance confidence***								
Ribeiro et al. 2015 [37]	53	80.29	FES	M = 63.6/100	GAI	M = 6.3/20	0.22	9
Zur et al. 2015 [54]	15	80.07	ABC	M = 87.6/100	SAST	M = 19.5/40	0.48	7
Yiu et al. 2012 [50]	98	67.64	ABC	M = 94.12/100	STAI-trait	M = 26.11/80	0.45	8
Valentine et al. 2011 [38]	153	81	ConfBAL	NA	HADS-A	NA	0.38	8
Anstey et al. 2009 [51]	717	NA	MFES	NA	GAS	NA	0.19	NA
Herman et al. 2009 [53]	252	76.3	ABC	M = 91.8/100	STAI-trait	M = 33.84/80	0.26	7
Williams et al. 2005 [49]	69	73.14	Modified ABC	M = 1226/1600	STAI-trait	M = 30.89/80	0.18	6
Gagnon et al. 2005 [48]	105	78.2	MFES	NA	HADS-A	NA	0.52	8
Burker et al. 1995 [45]	66	75	3-item FES	M = 4.20/18	SCL-90-R, Anxiety subscale	M = 0.12/NA	0.20	5
Tinetti et al. 1990 [24]	56	78	FES	M = 25.11/100	STAI-trait, cut-off of 37	Y = 25	0.36	8

*For this study, correlation coefficient was calculated from data presented in the article.

#### Quality of studies

Nineteen out of the twenty studies were assessed for quality. Anstey, Wood, Kerr, Caldwell and Lord’s study [51] was not assessed for quality because it was provided by Stephen Lord and Kim Delbaere as a complement to a set of unpublished analysis. This study was assessed only in regards to the psychometric validity of the scales used to measure anxiety and FRPCs.

The nineteen studies assessed have an average quality score of 7.58 out of 10. All studies report recruitment sources, inclusion and exclusion criteria, a description of statistical analysis of the association between anxiety and FRPCs and descriptive information about the participants. However, only two studies [i.e., [5,46]] report sample size calculations. The majority of studies describe the evaluation procedure in sufficient details, except for [53], [54] and [41]. Five out of nineteen studies report measuring fear of falling, but measure falls efficacy or balance confidence or vice-versa [i.e., [24,45,49,52,53]]. Most studies report their limitations, except for [45], [49] and [54].

Among the ten studies measuring fear of falling, only five use scales that demonstrated good reliability and validity [i.e., FES-I [22,55], Short FES-I [55,56] and SAFE [20]], while other studies use scales with insufficient psychometric information (see S1 Table). Among the ten studies measuring falls-efficacy or balance confidence, nine studies use scales that demonstrated good reliability and validity [i.e., FES [18,24], MFES [18,26], ABC [18,28] and CONFbal [18,29]], while other studies use scales with insufficient psychometric information (see S2 Table).

Among the twenty studies, a total of ten different scales are used to measure anxiety (see S3 Table). Among these scales, only four present an adequate amount of psychometric information for their use with older adults [i.e., Geriatric Anxiety Inventory [57,58], State-Trait Anxiety Inventory [58–61], the Hamilton Anxiety Rating Scale [58,62,63] and the Short Anxiety Screening Test [64]], even though most have at least some psychometric information demonstrating their adequacy of use with this population [58,65–72].

### Statistical analysis

**Fig 2:**
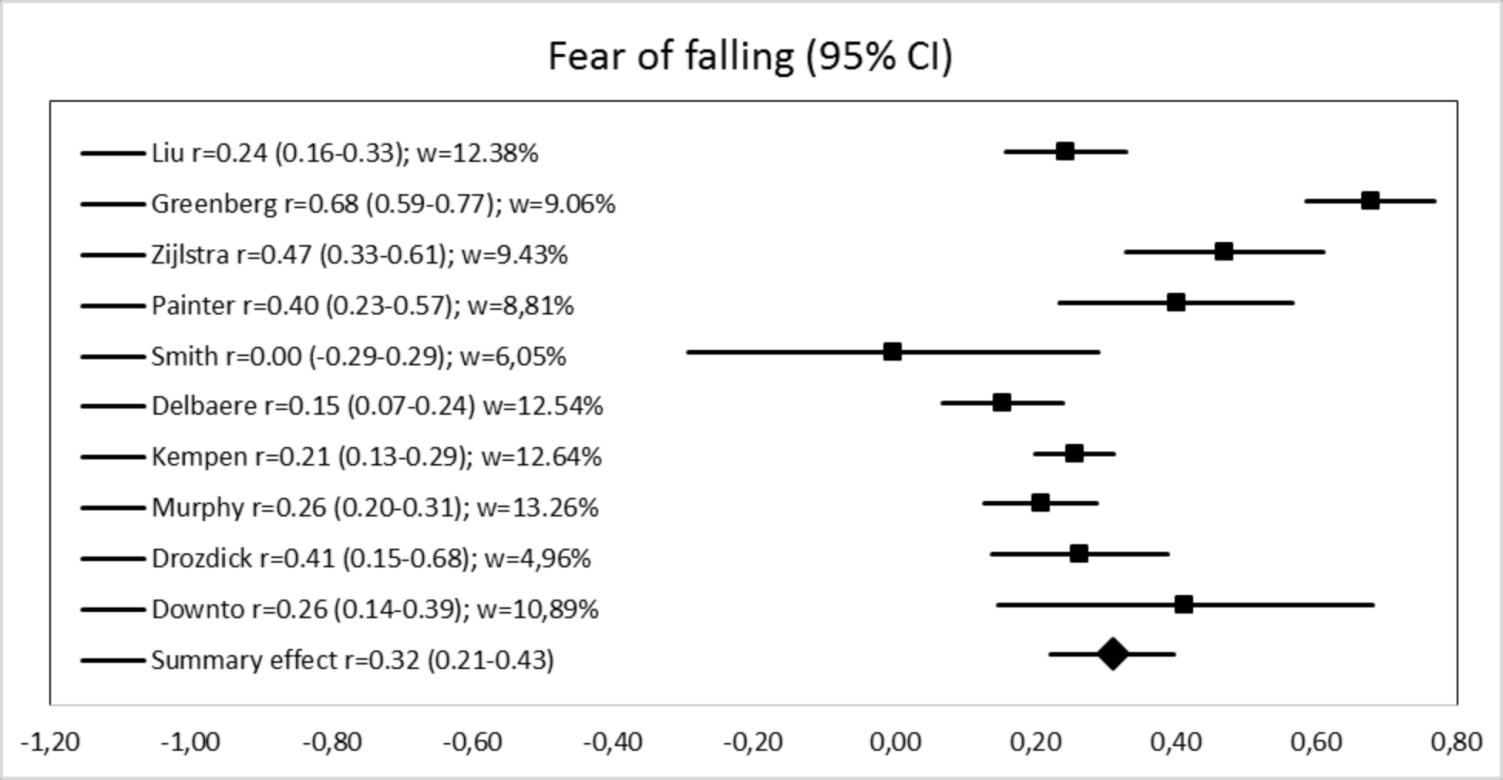
Forest plot of fear of falling studies.

**Fig 3:**
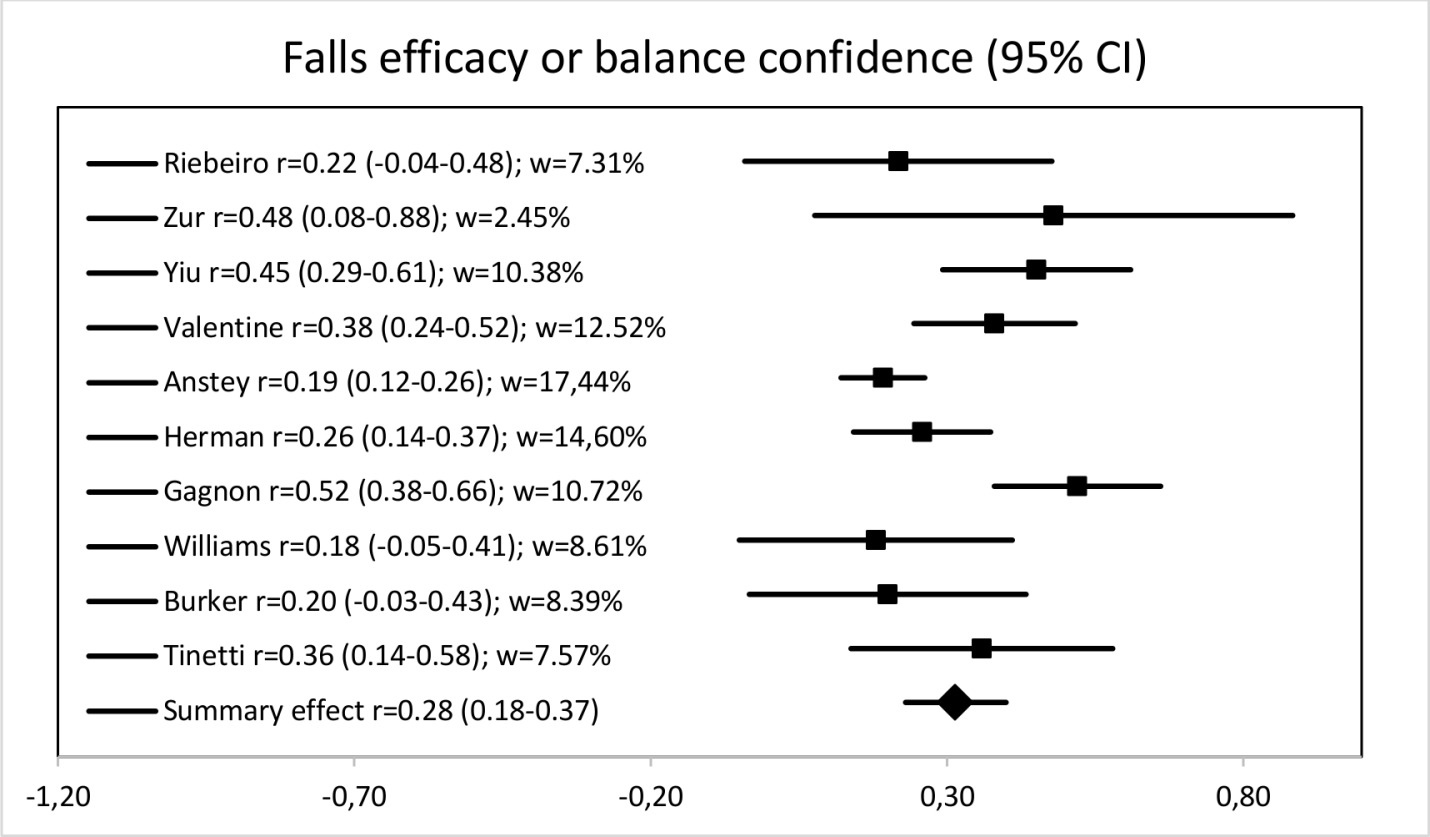
Forest plot of falls efficacy or balance confidence studies.

The summary effect (i.e., mean correlation with anxiety) with the random model is 0.32 (95% CI: 0.22–0.40), Z = 6.49, p < 0.001 for fear of falling and 0.31 (95% CI: 0.23–0.40), Z = 6.72, p < 0.001 for falls-efficacy or balance confidence, meaning that effect sizes are both medium, according to the conventions proposed by Cohen [73]. Anxiety explains 9.77% of fear of falling variance and 9.86% of falls-efficacy or balance confidence variance. However, correlations vary greatly across studies and heterogeneity is high for fear of falling studies according to Cochrane interpretation standards [35], with Q(9) = 53.60, p < 0.01 and I^2^ = 83.21%. Heterogeneity is moderate for falls-efficacy or balance confidence studies, with Q(9) = 22.81, p < 0.01 and I^2^ = 60.64% [35]. For fear of falling, correlations with anxiety range from 0.00 to 0.68 across studies and they range from 0.14 to 0.52 across falls-efficacy or balance confidence studies (see Figs 2 and 3).

Among fear of falling studies, heterogeneity is highly influenced by the inclusion of Greenberg’s study [46], in which all participants benefit from a nursing home level of care (and were nursing home eligible) because they suffer multiple chronic conditions, such as dementia or multiple sclerosis, but live in the community through the help of a specialized program. Since they live in the community, participants are technically eligible for inclusion in the meta-analysis, but since they have the characteristics of nursing home residents, they differ from the other participants. In cases where the results of a meta-analysis could be influenced by the arbitrary interpretation of predetermined inclusion criteria, The Cochrane Collaboration recommends undertaking a sensitivity analysis, which implies recomputing and interpreting the results with and without the studies for which the application of the inclusion criteria is not clear-cut [74]. Therefore, the mean effect size was recomputed without this study. Results show that correlation between anxiety and fear of falling is 0.26 (95% CI: 0.20–0.32), Z = 8.02, p < 0.001, meaning 6.59% of fear of falling variance is explained by anxiety. Heterogeneity is significant, but moderate according to The Cochrane Collaboration standards [35], with Q(8) = 20.16 p < 0.01 and I^2^ = 55.35%.

A Q-test for heterogeneity was conducted twice, once including Greenberg’s study [46] and once excluding it. Both results are not significant [Q(19) = 0.13, p = n.s. when including Greenberg’s study [46] and Q(18) = 0.94, p = n.s when excluding it], meaning that the two constructs do not appear to hold significantly different relationships with anxiety.

### Analysis of publication bias

**Fig 4:**
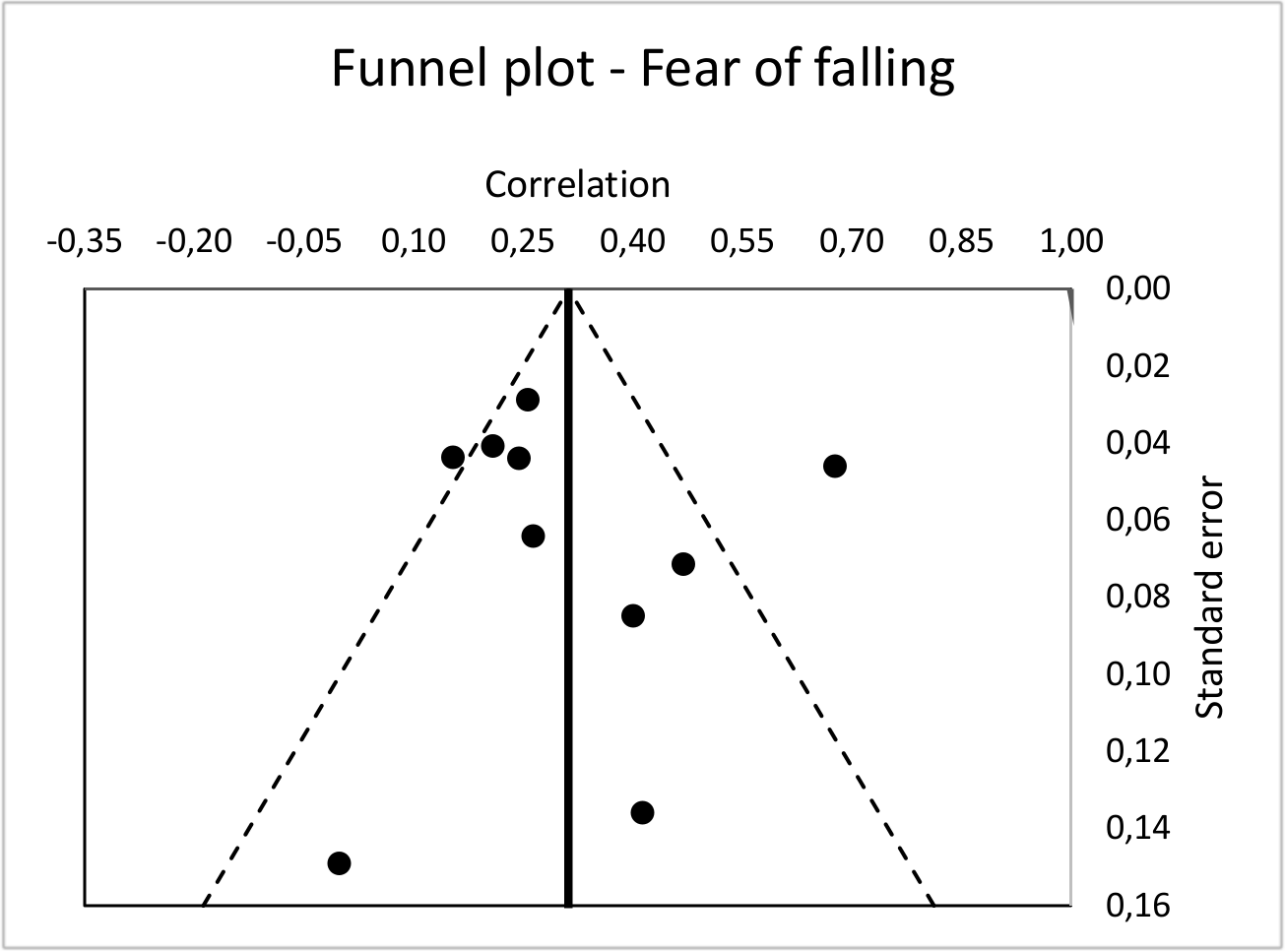
Funnel plot of fear of falling studies.

**Fig 5:**
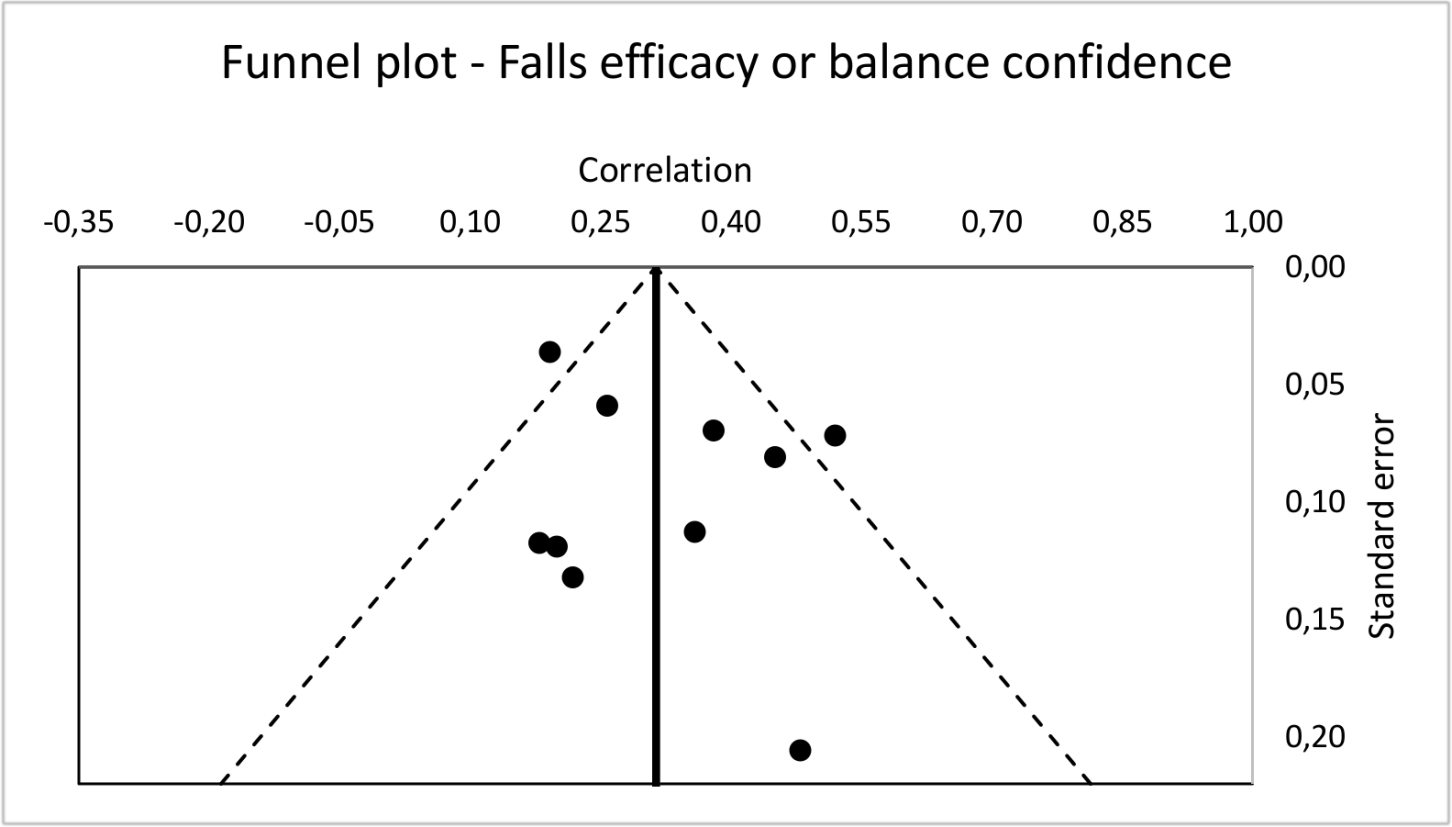
Funnel plot of falls-efficacy or balance confidence studies.

Funnel plots (see Figs 4 and 5) do not indicate the presence of a publication bias for fear of falling studies, nor for falls-efficacy or balance confidence studies. Rosenthal’s fail-safe n is 99.64 for the ten fear of falling studies and 107.55 for the ten falls-efficacy or balance confidences studies, well above their critical n of 60, thus indicating minimal risk of publication bias [34].

## Discussion

The present study is the first to provide a quantitative summary of the relationship between anxiety and FRCPs among the community-dwelling older adults. It demonstrates that anxiety is moderately and significantly associated with FRPCs in community-dwelling older adults and that the strength of this relationship does not vary depending on the use of 1) fear of falling and 2) falls-efficacy or balance confidence. This last finding is contrary to the conclusions of Denkinger et al.’s systematic review [12]. However, Denkinger et al.’s conclusions are based on three studies [12], while the results of the present analysis are based on twenty studies. The lack of significance of the Q-test for heterogeneity should not be interpreted as a lack of difference between the constructs: It only means that fear of falling and falls-efficacy hold similar relationships with anxiety among the 20 studies analyzed by the present meta-analysis. In order to derive such conclusions with other variables, such as falls, depression or activity restriction, further studies should be conducted. Providing evidence about whether fear of falling and falls-efficacy are different constructs is beyond the scope of the present meta-analysis and our conclusions should not be used to support the interchangeability of the terms or the measurement tools. Indeed, as reported by a psychometric study, the SAFE, which measures fear of falling, is moderately correlated with two falls-efficacy measures, the FES (r = 0.67) and the ABC scale (r = 0.66) [23]. However, the same study reports that the correlation between the two falls-efficacy measures is high (r = 0.86). This psychometric study and several reviews argue that falls-efficacy and fear of falling are related but distinct constructs and should be identified as such [4,14,18,75].

### Potential moderators related to methods and procedures of the included studies

The results of this meta-analysis indicate the presence of significant heterogeneity between the studies, even if the summary effect sizes are grouped by construct measured. Differences between studies may be partly due to differences in the methods and procedures employed by each study [36]. For example, some of the studies included in this meta-analysis used FRPC and anxiety measures with insufficient psychometric information. Even though there are many psychometrically sound scales for measuring FRPCs, single-item scales are still widely used in the literature. Although these measures are straightforward, quick and simple to use, concerns have been raised as to their long-term reliability [47]. In this meta-analysis, four of the 20 included studies used single-item scales. All of the studies using single-item scales measured fear of falling. In order to verify the impact of single-item measures on the results, a Q-test for heterogeneity was conducted between: 1) the four studies using single-item fear of falling scales and 2) the six other studies using multi-item scales for measuring fear of falling. The results were not significant [Q(9) = 0.99, p = n.s.], suggesting that the use of single-items scales or multi-items scales did not affect the relationship between fear of falling and anxiety.

Another possible moderator related to methods and procedures is the use of anxiety scales that were originally created for middle-aged adults and still lack psychometric information before their use can be recommended with older adults. Evaluating anxiety in older adults presents many challenges, such as the greater presence of various physical symptoms due to physical diseases, but also the greater use of physical complaints to express psychological concerns [57,58,67,76]. Therefore, it becomes harder to assess if some symptoms, such as insomnia or an upset stomach, are due to anxiety or medical conditions. It is of great relevance to question and verify the suitability of the scales for this particular population. In this meta-analysis, only two anxiety measures (Geriatric Anxiety Inventory and Short Anxiety Screening Test) were specifically designed for older adults [57]. Due to the limited number of included studies using these measures (n = 2), no moderation analysis could be conducted.

### Potential substantive moderators

Substantive moderators may also explain sources of heterogeneity among the included studies [36]. For example, FRPCs may be transient, especially in countries where rigorous winter conditions may impact the safety of outdoor walking surfaces. A prospective cohort study conducted in New York, USA, reported that 40% of incident fear of falling was temporary, which they defined as admitting fear of falling only once during a two-year period, during which participants were questioned every 2–3 months [77]. Weather conditions were not mentioned by the authors as a source of potential variance, but they cannot be ruled out.

Moreover, anxiety symptoms and anxiety disorders are heterogeneous, and different anxiety disorders have been known to relate differently with one another and with other conditions, such as depression [78]. They may also hold a different relationship with FRPCs. For example, FRPCs may hold a stronger relationship with GAD or a specific phobia of falls than with a panic disorder, even though both disorders come with anxiety symptoms. It would be interesting to study how FRPCs relate to specific anxiety disorders, such as GAD or specific phobia related to falls.

Furthermore, the common feature of anxiety disorders is the excessiveness of the fear or anxiety [7]. In order to determine if FRPCs are a potential manifestation of anxiety, the individual’s fall risk must be considered. This moderator could not be considered in the present meta-analysis, because it was generally not measured in the included studies. It is nonetheless interesting to note that Greenberg’s study [46] showed the strongest correlation between FRPCs and anxiety (r = 0.68), despite the fact that the participants had multiple chronic medical conditions, which rendered their FRPCs less likely to be excessive. Although Greenberg’s participants had many characteristics of elders living in institutions, a strong correlation was also observed in Gagnon’s study (r = 0.52), in which participants’ FRPCs were also less likely to be excessive, since they had all fallen in the past twelve months. Falls and limitations in activities of daily life (ADL) are strongly associated with FRPCs, as reported by a meta-analysis grouping more than 22,000 older adults [79]. A poor health status, such as frailty, a clinical syndrome characterized by a specific combination of poor health conditions (e.g., involuntary weight loss, low physical activity, or self-reported exhaustion) [80], has been associated with falls and anxiety [80–82]. Although the order of appearance of anxiety or FRPCs remains unclear [81], it would be interesting to study the relationships between FRPCs, fall risk, frailty and anxiety through further studies, in order to better understand if and how these variables are related with each other.

### Strengths and limitations

This is the first study to provide a quantitative summary of the association between FRCPs and anxiety among community-dwelling elderly. Efforts were made to include unpublished data and limit potential publication biases. Even though some potentially relevant studies could not be included, due, for example, to the impossibility to contact authors or language limitations, the possibility of a publication bias was categorized as “low” upon analysis of funnel plots and Rosenthal’s fail-safe n. Another limitation of the present study is that it could not account for some of the previously mentioned moderators, because the information was not available in the included studies. Future studies should examine, as Delbaere et al. [5], the relationship between anxiety and FRPCs while considering the participant’s actual risk of falls.

### Choosing among FRPC scales

There is no consensus regarding whether it is better to measure fear of falling or falls-efficacy. It remains nonetheless important to clearly identify which of these two constructs is being measured [4,14,18,75]. Moreover, within the two categories of constructs, some scales have demonstrated stronger psychometric properties than others. Among the scales measuring fear of falling, the FES-I, the Short FES-I and the SAFE have all demonstrated good psychometric properties for community-dwelling older adults (see S1 Table). All three scales measure concern or worry about falling during basic and more demanding activities, which are both important to assess among autonomous older adults [20,22,56]. Because there is currently more information about the reliability of the FES-I and the Short FES-I [55], these scales should be preferred over the SAFE. In addition, the FES-I and the Short FES-I have validated cut-off points for distinguishing between low, moderate and high levels of concern [55].

Among the scales measuring falls-efficacy or balance confidence, the FES, MFES, ABC and CONFbal have all demonstrated good psychometric properties for community-dwelling older adults (see S2 Table). The FES and the ABC have been the most widely used and extensively validated [18]. Since the FES measures confidence during basic activities only (e.g., take a bath or shower; get in and out of bed) and the ABC scale measures confidence during both basic activities and more demanding ones (e.g., walking in a crowded mall), the ABC scale can be more useful to evaluate falls-efficacy among autonomous older adults living in the community [28].

## Conclusion

This study demonstrates the relevance of studying anxiety in the context of FRPCs. Future studies should focus on analysing the relationship between anxiety and FRPCs when considering fall risk and on differentiating those with excessive concerns from those with realistic concerns. As suggested by Murphy, Williams and Gill [47], it is possible that for some older adults, FRPCs are a manifestation of a more generalized anxiety problem such as GAD, and represent one of the individual’s worry themes. Future studies should assess the relationship between GAD and FRPCs. In addition, the prevalence of specific phobia related to falls among individuals with FRPCs is unknown and merits some attention. Finally, future studies and clinicians should also make sure that the scales used to measure FRPCs and anxiety have demonstrated their reliability and validity with older adults.

## Supporting Information

S1 File Full search strategy. (DOCX)

S1 Table Fear of falling scales. (DOCX)

S2 Table Falls efficacy or balance confidence scale. (DOCX)

S3 Table Anxiety scales. (DOCX)

S4 Table PRISMA 2009 Checklist. (DOC)
