# Supplementary material for: Fall-Related Psychological Concerns and Anxiety among Community-Dwelling Older Adults: Systematic Review and Meta-Analysis
Source: PLoS One. 2016 Apr 4;11(4):e0152848. doi: 10.1371/journal.pone.0152848 (PMC4820267; doi:10.1371/journal.pone.0152848)
Supplement: S1 File — (DOCX) [file pone.0152848.s001.docx]

**S1 File. Full search strategy**

1. PsycINFO

Anxiety OR Anxiety Disorders (abstract) AND (« Fear of falling » or « Falls efficacy » or « Balance confidence » or « ptophobia » or “fall-related concern”) (abstract).

June 9^th^ 2015: 66 results

1. Web of Science

Anxiety or "anxiety disorder*" in TOPIC

AND "fear of fall*" or "balance confidence" or "falls efficacy" or ptophobia or "fall-related concern*" in TOPIC

June 9^th^, 2015: 143 results

1. Web of Science

Anxiety or "anxiety disorder*" in TOPIC

AND "fear of fall*" or "balance confidence" or "falls efficacy" or ptophobia or "fall-related concern*" in TITLE

June 9^th^, 2015: 72 results

1. MEDLINE (with FullText (EBSCO) (XML))

Anxiety in AB (Abstract)

AND "fear of falling" OR "balance confidence" OR "falls efficacy" OR ptophobia OR “fall-related concern” in AB

June 9th, 2015 : 137 (83 without duplicates eliminated by MEDLINE)

1. SCOPUS

(TITLE-ABS-KEY(anxiety) AND TITLE-ABS-KEY(“fear of falling” OR “balance confidence” OR “falls efficacy” OR “ptophobia” OR “fall-related concern”))

June 9^th^, 2015: 136 results

1. PubMed

(anxiety[Title/Abstract]) AND ("fear of falling"[Title/Abstract] OR "balance confidence"[Title/Abstract] OR "falls efficacy"[Title/Abstract] OR "ptophobia"[Title/Abstract] or "fall-related concern"[Title/Abstract])

June 9^th^, 2015: 98 results

1. CINAHL Plus with Full Text

Anxiety in AB (Abstract)

AND "fear of falling" OR "balance confidence" OR "falls efficacy" OR ptophobia OR “fall-related concern” in AB

June 9^th^, 2015: 45 results
