# Supplementary material for: Fall-Related Psychological Concerns and Anxiety among Community-Dwelling Older Adults: Systematic Review and Meta-Analysis
Source: PLoS One. 2016 Apr 4;11(4):e0152848. doi: 10.1371/journal.pone.0152848 (PMC4820267; doi:10.1371/journal.pone.0152848)
Supplement: S1 Table — (DOCX) [file pone.0152848.s002.docx]

**S1 Table. Fear of falling scales**

| ***Scale*** | ***Used by*** | ***Psychometric properties (for elders)*** |
| --- | --- | --- |
| Falls-efficacy scale International (FES-I) [22] | Greenberg [46], Delbaere et al [5] | Internal reliability (Cronbach’s alpha)=0.96 and test-retest reliability=0.96 [22]; convergent and predictive validity with fall risk factors, such as gender age, multiple falls [55] |
| Short FES-I [56] | Zijlstra et al [41] | Internal reliability (Cronbach’s alpha)=0.92 and test-retest reliability=0.87 [55]; convergent and predictive validity with fall risk factors, such as gender, age, multiple falls [55,56] |
| Chinese FES-I* | Liu [52] | Not available |
| Survey of activity and Fear of falling in the Elderly (SAFE) [20] | Painter et al [42], Smith et al [43] | Internal reliability (Cronbach’s alpha)=0.91, convergent validity (correlation with the FES)=-0.76 and correlation with fall risk factors, such as gender, age and falls [20] |
| Are you afraid of falling? 5-point Likert scale | Kempen et al [39] | Not available |
| Are you afraid of falling?  Y/N | Murphy et al [47] | Test-retest reliability (kappa statistic)=0.66 [24] |
| Single question on severity of FoF (no details) | Drozdick and Edelstein [44] | Not available |
| Do you limit your activities because of Fof? Y/N | Downton and Andrews [40] | Not available |

*The author refers to an article that has validated a Chinese version of the FES, a 10-item scale measuring self-efficacy, on a scale of 0-100, but describes having used a Chines version of the FES-I, a 16-item scale measuring concern about falls on a scale of 16-64.
