# Supplementary material for: Fall-Related Psychological Concerns and Anxiety among Community-Dwelling Older Adults: Systematic Review and Meta-Analysis
Source: PLoS One. 2016 Apr 4;11(4):e0152848. doi: 10.1371/journal.pone.0152848 (PMC4820267; doi:10.1371/journal.pone.0152848)
Supplement: S2 Table — (DOCX) [file pone.0152848.s003.docx]

**S2 Table. Falls-efficacy or balance confidence scales**

| ***Scale*** | ***Used by*** | ***Psychometric properties (for elders)*** |
| --- | --- | --- |
| Falls Efficacy Scale (FES) [24] | Ribeiro and Santos [37], Tinetti et al [24] | Good reliability and validity. See Jorstad et al. [18] for a summary of psychometric studies. |
| Modified Falls Efficacy Scale (MFES) [26] | Gagnon et al [48], Anstey et al [51] | Good reliability and validity. See Jorstad et al. [18] for a summary of psychometric studies. |
| 3-item from the Falls Efficacy Scale [45] | Burker et al [45] | Internal reliability (Cronbach’s alpha)=0.93 [45] |
| Activities-specific Balance and Confidence scale (ABC) [28] | Williams et al [49]*, Yiu et al [50], Herman et al [53], Zur et al [54] | Good reliability and validity. See Jorstad et al. [18] for a summary of psychometric studies. |
| Confidence in maintaining Balance scale (CONFbal) [29] | Valentine et al [38] | Good reliability and validity. See Jorstad et al. [18] for a summary of psychometric studies. |

*Williams et al [49] use the ABC scale, but score it with a 21-point horizontal box scale, with a total score ranging from 0 to 1600 (max score = 100 for each of the 16 items).
