# Supplementary material for: Fall-Related Psychological Concerns and Anxiety among Community-Dwelling Older Adults: Systematic Review and Meta-Analysis
Source: PLoS One. 2016 Apr 4;11(4):e0152848. doi: 10.1371/journal.pone.0152848 (PMC4820267; doi:10.1371/journal.pone.0152848)
Supplement: S3 Table — (DOCX) [file pone.0152848.s004.docx]

**S3 Table. Anxiety scales**

| ***Scale*** | ***Used by*** | ***Psychometric properties (for elders)*** |
| --- | --- | --- |
| Geriatric Anxiety Inventory (GAI) [57] | Ribeiro et al [37] | Internal reliability (Cronbach’s alpha=0.91), test-retest reliability among a geriatric psychiatry sample (r=0.91), inter-rater reliability among a geriatric psychiatry sample (r=0.99), concurrent validity (significant correlations with GAS, STAI, Beck Anxiety inventory, and Penn State Worry Questionnaire), ability to differentiate older adults with an anxiety disorder from those without [57]. A review by Therrien and Hunsley [58] concludes that current evidence supports the use of the GAI with older adults. |
| Hamilton Anxiety Rating Scale (HARS) [62] | Painter et al [42] | Internal reliability (Cronbach’s alpha between 0.76 and 0.86, depending on studies), inter-rater reliability (r=0.81-0.95, depending on studies), good ability to differentiate between older adults with GAD and older adults with no anxiety disorders [58] and  good ability to correctly diagnose GAD [63]. However, a review by Therrien and Hunsley [58] concludes it is not an optimal choice for older adults, because of concerns about its discriminant validity and a lack of norms for this population. |
| Chinese General Anxiety Disorder Questionnaire – 7 (C-GAD-7) | Liu [52] | Information not available in French or English |
| State-Trait Anxiety Inventory (STAI) [59] | Tinetti et al [24], Smith et al [43], Drozdick and Edelstein [44], Murphy et al [47], Williams et al [49], Yiu et al [50], Herman et al [53] | Internal reliability (Cronbach’s alpha=0.92) [59], test-retest reliability (r=0.74) [60], sensitivity= 0.82 and specificity=0.88 [61]. A review by Therrien and Hunsley [58] concludes that the STAI is not an optimal choice for older adults, because of mixed results regarding its validity. |
| Goldberg Anxiety Scale (GAS) [66] | Delbaere et al [5], Anstey et al [51] | Internal reliability (Cronbach’s alpha=0.74) [67], low kappa values with self-reported doctor diagnosis, medication usage and SF-36 subscale in a sample of women aged 75-8 (kappa ranging from -0.13 to 0.28) [68]. A review by Therrien and Hunsley [58] concludes that the GAS should not be used with older adults, due to insufficient psychometric support. |
| Hospital Anxiety and Depression Scale – Anxiety subscale (HADS-A) [69] | Valentine et al [38], Kempen et al [39], Zijlstra et al [41], Gagnon et al [48] | Internal reliability (Cronbach alpha=0.82) in a community sample of older adults, correlation with the HARS (r=0.57) in a sample of elderly psychiatric inpatients and outpatients [70]. A review by Therrien and Hunsley [58] considers that the HADS-A should not be used for assessing anxiety among older adults, due to the lack of psychometric studies. |
| General Health Questionnaire - Anxiety subscale (GHQ) [71] | Downton and Andrews [40] | Internal consistency (Cronbach’s alpha=0.82) [59], no test-retest reliability studies, but ability to differentiate between older adults with or without mental disorders [58]. A review by Therrien and Hunsley [58] recommends considerable caution with the GHQ due to the lack of psychometric information for assessing anxiety with older adults. |
| Symptom Checklist 90-R (SCL-90-R), Anxiety subscale [72] | Burker et al [45] | Internal consistency (Cronbach’s alpha=0.90) [59] but no other psychometric information with older adults [58]. A review by Therrien and Hunsley [58] considers that the SCL-90-R should not be used for assessing anxiety among older adults, due to the lack of psychometric studies. |
| Diagnosis of anxiety, based on International Classification of Diseases (ICD-9) | Greenberg [46] | Not available |
| Short Anxiety Screening Test (SAST) [64] | Zur et al [54] | Internal consistency (Cronbach’s alpha=0.70), test-retest interrater reliability (r=0.73), interrater reliability (kappa=0.71), sensitivity of 75.4% for detecting anxiety diagnosis, specificity of 78.7% in a sample of elderly medical inpatients and outpatients [64]. |
